# Prevalence of unintended pregnancy and its associated factors: Evidence from six south Asian countries

**DOI:** 10.1371/journal.pone.0245923

**Published:** 2021-02-01

**Authors:** Alamgir Sarder, Sheikh Mohammed Shariful Islam, Maniruzzaman, Ashis Talukder, Benojir Ahammed

**Affiliations:** 1 Statistics Discipline, Khulna University, Khulna, Bangladesh; 2 Institute for Physical Activity and Nutrition (IPAN), School of Exercise and Nutrition Sciences, Deakin University, Melbourne, Australia; University of Cape Coast, GHANA

## Abstract

**Aim:**

Unintended pregnancy is a significant public health concern in South Asian countries because of its negative association with the socioeconomic and health outcomes for both children and mothers. The present study aimed to explore the prevalence of unintended pregnancy and explore its determinants among women of reproductive age in six South Asian countries.

**Methods:**

Nationwide latest demography and health survey data from six South Asian countries, including Bangladesh (2014), Pakistan (2017–2018), Nepal (2016), Afghanistan (2015), Maldives (2016–2017) and India (2015–2016) were pooled for the present study. Multivariate analysis was performed to explore the association between unintended pregnancy and its associated factors.

**Results:**

Amongst the total women (n = 41,689), overall, 19.1% pregnancies were reported as unintended (ranging from 11.9% in India to 28.4% in Bangladesh). The logistic regression model showed that younger women (15–19 years) had 1.42 times higher chance of unintended pregnancies. The odds of unintended pregnancies was 1.24 times higher for poorest women and 1.19 times higher for poorer women. Further, urban women (aOR = 0.70, 95% CI = 0.50–0.80), women having no children (aOR = 0.10, 95% CI = 0.09–0.12), smaller (≤4) family (aOR = 0.72, 95% CI = 0.67–0.78), those who intent to use contraceptive (aOR = 0.72, 95% CI = 0.60–0.86), currently living with partner (aOR = 0.90, 95% CI = 0.81–0.99), first cohabitation in teenage (≤19 years) (aOR = 0.85, 95% CI = 0.78–0.92) were less likely to report unintended pregnancies.

**Conclusions:**

This study has showed that women’s age, wealth index, place of residence, number of children, family size, the intention of contraceptive use, living with a partner, and first cohabitation age are essential determinants of unintended pregnancy. These factors should be considered when trying to reduce unintended pregnancy in six South Asian countries. However, there is a need to improve health education, counselling, skills-building, sex education, modern contraceptive use and its access in this region. Intervention programs regarding reproductive health and policies are warranted to reduce rates of unintended pregnancy in South Asian countries.

## Introduction

Unintended pregnancy (UP) is a global public health problem and can affect any sexually active women. Unintended pregnancy is defined when women did not desire to become pregnant at that time or at any time in the future [1,2]. Unintended pregnancies have a negative impact on women’s personal life, their families, and society. Globally, 74 million women had unintended pregnancies in low and middle-income countries, and every year around 25 million unsafe abortions and 47 thousand maternal deaths occur. A remarkable extent of unintended pregnancies brings about premature birth and another unfavorable pregnancy result [3,4]. It is the consequences of a wide range of factor including non-use of contraception, contraceptive discontinuation, contraceptive defeat, inconsistency and inaccurate use of contraception, and lack of awareness regarding family planning [5]. Globally, unintended pregnancy had been decreased in high-income countries compared with low- and middle-income countries [6]. The unintended pregnancy remained a global public health problem and substantially higher in low- and middle-income regions. Approximately 53.8 million unintended pregnancies occurred each year in Asia. About 5.4% of women aged 15–44 years were suffered from unintended pregnancies during the year 2010–2014 in Asia [7]. South Asian countries, which includes the sub-Himalayan and neighboring countries of Bangladesh, Pakistan, Nepal, Afghanistan, Maldives, India, Sri Lanka and Bhutan are low- and middle-income countries [8]. India, the prevalence of unintended pregnancy was not demonstrated a lot of variety or to a narrow dosage had been stale during last one decade [9]. Around one-fourth of the women in India was reported that their pregnancy was unintended in each of the three rounds of National Family Health Surveys (NFHS) [10]. In 2012, a study revealed that about 29% of pregnancies were unintended and the frequency of unintended pregnancy was higher among older, poor, and less educated women of rural area in Bangladesh [11]. However, the unintended pregnancy was decreased 4% from the year 1993 to 2011 in Bangladesh [12]. Among South Asian countries, Nepal has recorded the highest (50%) unintended pregnancy [13]. A hospital-basedcross-sectional survey in Pakistan reported that 38.2% of pregnancies were unintended [5]. Another study in Afghanistan showed that 36.9% of unplanned pregnancies were common among women [14]. In addition, poor knowledge in contraceptive use, low socioeconomic status, contraceptive failure, sexual violence, shortage in contraceptive supply, unmarried status, age, religion, number of children, residence, wealth index, the intension of contraceptive use and first cohabitation age influences the unintended pregnancies [6,7]. However, few studies found that inconsistent and incorrect condom use, contraceptive failure, and lack of knowledge on emergency contraception were treated as the reason for current unintended pregnancy in some other countries [15–17].The unsafe abortion, maternal death, malnutrition, mental illness and vertical transmission of HIV to children were the most severe consequences of unintended pregnancy [18–20]. These had negative impacts on women’s quality of life, and increase the economic cost of families as well as increases the mental stress of the women that were the causes of maternal and neonatal morbidity and mortality [5].

Most of the previous studies were conducted only on the prevalence and determinants of unintended pregnancy using cross-sectional data of individual countries in South Asia. A country-specific investigation was conducted in Bangladesh [11,12], Pakistan [5,21], Nepal [13], Maldives [22] and India [23,24] to investigate the predictors of unintended pregnancy. Previously an experiment was conducted in 2015 to assess the association between intimate partner violence (IPV) and unintended pregnancy in South Asia [8]. To the best of our knowledge, there are no studies investigating the phenomenon across South Asian countries. Therefore, the main objectives of this study were to (i) identify the prevalence of unintended pregnancy among women in six South Asian countries aged 15–49 years; (ii) examine the association between sociodemographic and behavioral characteristics and unintended pregnancy; and (iii) examine the impact of sociodemographic and behavioral factors on unintended pregnancy. This analysis will utilize pooled data drawn from the demographic and health survey (DHS) to address the objectives of this study. The results of this study will be useful to develop programs to improve reproductive health program to reduce the unintended pregnancy in South Asian countries.

## Methods

### Ethical approval

This study was performed by secondary data analysis of data collected from MEASURE demography health and survey (DHS). The DHS surveys obtained ethical clearance from the Ethics Committee of ORC Macro Inc., and the Ethics Boards of Ministry of Health of the considered six South Asian countries. This survey confirmed international ethical standards and during each of the surveys, either written or verbal consent, was provided by the women. The details of ethics approval of six South Asian countries is described elsewhere [25–30].

### Data sources

DHS is a large scale nationally representative cross-sectional survey of households that collects data on population, health, HIV, and nutrition through more than 400 surveys in over 90 countries [31]. DHS in different countries collects data of all individual ever-married women aged 15 to 49 years in the household using personal interviews by trained interviewers and a well-designed questionnaire. The survey used a two-stage stratified sampling technique, sampling within administrative areas [32]. The detail of the study design was described elsewhere [33]. This study was a cross-sectional study, used a pooled dataset from current Demographic and Health Surveys (DHS). Data were limited to the most recent standard DHS from six countries in South Asia. The six countries are Bangladesh (2014), Pakistan (2017–2018), Nepal (2016), Afghanistan (2015), Maldives (2016–2017) and India (2015–2016). The principal reason for choosing these six countries is that data on some of the sociodemographic, and behavioral characteristics of interest were only available in these six countries. These six countries had current DHS information and all the factors of interest for this study. Some South Asian nations were excluded from this study because of lacking related data. The analysis in this study is limited to currently ever-married women aged 15 to 49 years (N = 42,578). Further 889 women were excluded due to missing data and the analysis included 41,689 observations. All dataset is available to the public online [34].

### Dependent variable

The primary outcome variable of the study was pregnancy intentions status. “Pregnancy intentions” was accounted for the dependent variables for this study which emerged from the inquiry concerning whether women intended their present pregnancy or not. The DHS document has a question for women as "current pregnancy wanted" and it has three responses, namely: ‘then’, ‘later’ and ‘not at all’. For simplicity we have coded these three responses as follows: ‘then’ for intended (0); ‘later or not at all’ for ‘unintended (1)’ based on the definition of unintended pregnancy [35].

### Explanatory variables

A set of categorical explanatory variables was selected to fit the two individual regression model. At the model-1, we considered the only country of origin as an independent variable. Based on the several studies twelve explanatory variables were considered as independent variables in model-2, namely age (15–19, 20–24, 25–29, 30–34, 35–39, 40–49), residence (urban, rural), educational level (no institutional education, primary, secondary & higher), wealth index (poorest, poorer, middle, richer, richest), religion (Islam, Hinduism, others), household head (male, female), the number of children (no children, 1–2 children, 3 or more), family size (≤ 4, > 4), the intention of contraceptive use (intends to use, does not intend to use), current residence with a partner (living with a partner, staying elsewhere), first cohabitation age (≤ 19 years, > 19 years) and country of origin (Bangladesh, Pakistan, Nepal, Afghanistan, Maldives, India). These variables were selected as there was a significant association with pregnancy intention and had been reported as predictors of unintended pregnancy [6,7,36–38].

### Statistical analysis

Simple descriptive analysis and bivariate and multivariate statistical analyses were performed in this study. Descriptive analysis was commenced to describe the frequency and percentage distribution. Bivariate analysis was used to examine the association between the unintended pregnancy and selected independent variables. Multivariable binary logistic regression analyses were conducted to assess the effect of different sociodemographic and behavioral factors on unintended pregnancy. Logistic regression (in Model 1) examines the effect of the country on unintended pregnancy. Multivariable logistic regression (in Model 2) examines the effect of selected sociodemographic and behavioral factors on unintended pregnancy. The results of the logistic regression analysis were presented using adjusted odds ratios (aORs) along with 95% confidence intervals. The high-risk factors were identified based on p-value (p<0.05). All the statistical analyses were performed in IBM SPSS v20 (SPSS Inc, Chicago, IL).

## Results

### Background characteristics of participants

**Fig 1:**
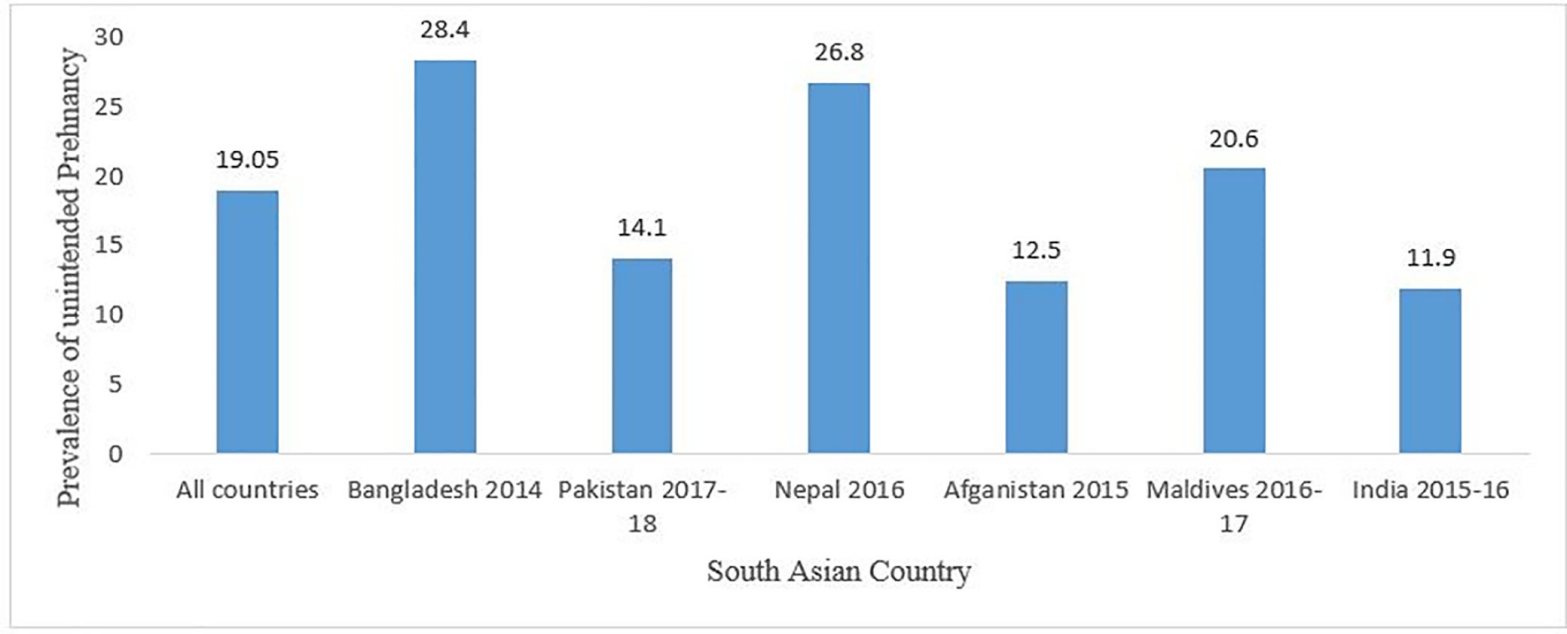
Prevalence of unintended pregnancy in South Asian countries.

Fig 1 presented the distribution of unintended pregnancies of six South Asia countries. Overall, 19.1% of unintended pregnancies were found from six South Asian countries. The prevalence of unintended pregnancies was ranged from 11.9% in India to 28.4% in Bangladesh. The background characteristics of the study participants and bivariate analysis of sociodemographic and behavioral variables with unintended pregnancy in six South Asian countries were presented in Table 1. Considering the women age, most of the respondents were from 20–24 years (39.3%) age group. The study women were predominantly rural, with only one-fourth of the women residing in an urban area. Whereas, the percentage of the study women was gradually decreased with increasing the educational level. Nearly half of the women were from the poor and poorest family whereas one-third of the women were from rich and richest family. Majority of the study women were intended to use contraceptive (75.9%), and they had 1–2 children (43.7%). It was observed that the proportion of unintended pregnancy increases with increasing age and number of children of the women. Subsequently, it was also observed that the rate of unintended pregnancy decreases with increasing the educational level and wealth index of the women. In this study, the rate of unintended pregnancy also higher among Hindu women (15.0%), women’s having >4 family members (14.4%), intent to contraceptive use (14.0%), life partner living/staying elsewhere (13.8%) and first cohabitation age ≤19 years (14.2%). Except for household head of the respondents, all the selected explanatory variables were found significantly (p < 0.05) associated with an unintended pregnancy.

**Table 1:** Background characteristics of the study participants and bivariate analysis of sociodemographic and behavioral variables with unintended pregnancy in six South Asian countries.

			Pregnancy status		Chi-square value (p-value)
Variables	N	%	Intended, n (%)	Unintended, n (%)	
Total	41,689				
**Age (in years)**					269.2 (<0.001)
15–19	4861	11.7	4350(89.5)	511(10.5)	
20–24	16375	39.3	14515(88.6)	1860(11.4)	
25–29	12356	29.6	10775(87.2)	1581(12.8)	
30–34	5112	12.3	4355(85.2)	757(14.8)	
35–39	2228	5.3	1817(81.6)	411(18.4)	
40–49	757	1.8	554(73.2)	203(26.8)	
**Residence**					4.4 (0.019)
Urban	10206	24.5	8964(87.8)	1242(12.2)	
Rural	31483	75.5	27402(87.0)	4081(13.0)	
**Educational level**					168.1 (<0.001)
No education	16833	40.4	14304(85.0)	2529(15.0)	
Primary	16628	39.9	14604(87.8)	2024(12.2)	
Secondary & higher	8228	19.7	7458(90.6)	770(9.4)	
**Wealth index**					128.9 (<0.001)
Poorest	9662	23.2	8178(84.6)	1484(15.4)	
Poorer	9914	23.8	8556(86.3)	1358(13.7)	
Middle	8568	20.6	7546(88.1)	1022(11.9)	
Richer	7359	17.7	6508(88.4)	851(11.6)	
Richest	6186	14.8	5578(90.2)	608(9.8)	
**Religion**					77.3 (<0.001)
Islam	32868	78.8	28647(87.2)	4221(12.8)	
Hinduism	5330	12.8	4530(85.0)	800(15.0)	
Others	3491	8.4	3189(91.3)	302(8.7)	
**Household head**					2.2 (0.071)
Male	37302	89.5	32508(87.1)	4794(12.9)	
Female	4387	10.5	3858(87.9)	529(12.1)	
**Number of children**					1501.7 (<0.001)
No children	14636	35.1	13836(94.5)	800(5.5)	
1–2 children	18201	43.7	15687(86.2)	2514(13.8)	
3 or more children	8852	21.2	6843(77.3)	2009(22.7)	
**Family size**					217.9 (<0.001)
≤4	13111	31.4	11904(90.8)	1207(9.2)	
>4	28578	68.6	24462(85.6)	4116(14.4)	
**Intention of contraceptive use**					181.9 (<0.001)
Intends to use	31640	75.9	27207(86.0)	4433(14.0)	
Does not intend to use	10049	24.1	9159(91.1)	890(8.9)	
**Current residence with partner**					4.0 (<0.024)
Living with partner	37500	90.0	32753(87.3)	4747(12.7)	
Staying elsewhere	4189	10.0	3613(86.2)	576(13.8)	
**First cohabitation age**					117.6 (<0.001)
≤19 years	25095	60.2	21529(85.8)	3566(14.2)	
>19 years	16594	39.8	14837(89.4)	1757(10.6)	

### Multivariate logistic regression analysis of unintended pregnancy

*Table 2* presented the results of the logistic regression analysis to shows the effect of unintended pregnancy among six South Asian countries women by background characteristics. Unadjusted effect of the country on unintended pregnancies was measured in Model 1. Among the six south Asian countries, Bangladesh (OR = 2.94, 95% CI = 2.56–3.37) had significant highest odds of unintended pregnancy among women followed by Nepal (OR = 2.70, 95% CI = 2.22–3.29), Maldives (OR = 1.92, 95% CI = 1.44–2.56) and Pakistan (OR = 1.21, 95% 95% CI = 1.06–1.40) compared with India as a reference country. In Model 2, after adjusting the socio-demographic and behavioral characteristics, an almost similar result was found for the country of the region. The results indicated that Bangladesh (aOR = 2.97, 95% CI = 2.57–3.45), Nepal (aOR = 2.58, 95% CI = 2.09–3.19), and Maldives (aOR = 2.42, 95% CI = 1.78–3.28) had significantly higher odds of unintended pregnancy among the women, but Pakistan (aOR = 0.79, 95% CI = 0.68–0.92) and Afghanistan (aOR = 0.57, 95% CI = 0.51–0.63) had significantly lower odds of unintended pregnancy compared to women of India.Women aged 15–19 years had higher odds (aOR = 1.42, 95% CI = 1.13–1.78) and all others age categories had lower odds of unintended pregnancies compared with women aged 40–49 years. Urban women were less likely to commit to unintended pregnancy, as compared with rural women (aOR = 0.70, 95% CI = 0.50–0.80). Highest odds occurred among Poorest (aOR = 1.24, 95% CI = 1.09–1.42) and poorer (aOR = 1.19, 95% CI = 1.05–1.35) women respectively, compared to richest.Women having religious faith in Islam (aOR = 1.52, 95% CI = 1.34–1.73) and Hinduism (aOR = 1.80, 95% CI = 1.52–2.04) had higher odds of unintended pregnancies compare with other religious women. The women had no children (aOR = 0.10, 95% CI = 0.09–0.12) and 1–2 children (aOR = 0.40, 95% CI = 0.36–0.44) reported that they had lower odds of unintended pregnancy, as compared with the women who had at least three children. It was also interesting to find that small family (≤4) were less likely to have unintended pregnancy (aOR = 0.72, 95% CI = 0.67–0.78) than large family (>4). Compared to the women does not intend to use a contraceptive; the odds of unintended pregnancies were 0.72 times lower for the women intend to use contraceptive (aOR = 0.72, 95% CI = 0.60–0.86). The women who lived with her partner recorded the lower odds of unintended pregnancy compared with women’s partner staying elsewhere (aOR = 0.90, 95% CI = 0.81–0.99). First cohabitation age ≤19 years of women had fewer likelihoods of unintended pregnancy (aOR = 0.85, 95% CI = 0.78–0.92), as compared to their counterpart.

**Table 2:** Multivariate logistic regression analyses showing the effect of unintended pregnancy among six south Asian countries women by background characteristics.

Variables	Sample size	Model I OR [95% CI]	p-value	Model II aOR [95% CI]	p-value
**Country**					
Bangladesh	1076	2.94[2.56–3.37]	<0.001	2.97[2.57–3.45]	<0.001
Pakistan	1729	1.21[1.06–1.40]	0.006	0.79[0.68–0.92]	0.002
Nepal	519	2.70[2.22–3.29]	<0.001	2.58[2.09–3.19]	<0.001
Afghanistan	6260	1.06[0.97–1.15]	0.189	0.57[0.51–0.63]	<0.001
Maldives	286	1.92[1.44–2.56]	<0.001	2.42[1.78–3.28]	<0.001
India	31819	Ref		Ref	
**Age (in years)**					
15–19				1.42[1.13–1.78]	0.002
20–24				0.89[0.74–1.08]	0.248
25–29				0.63[0.52–0.75]	<0.001
30–34				0.55[0.45–0.66]	<0.001
35–39				0.64[0.52–0.78]	<0.001
40–49				Ref	
**Residence**					
Urban				0.70[0.50–0.80]	0.034
Rural				Ref	
**Educational level**					
No institutional education				0.93[0.83–1.04]	0.183
Primary				0.97[0.86–1.06]	0.473
Secondary & higher				Ref	
**Wealth index**					
Poorest				1.24[1.09–1.42]	0.001
Poorer				1.19[1.05–1.35]	0.006
Middle				1.09[0.98–1.23]	0.154
Richer				1.10[0.97–1.23]	0.132
Richest				Ref	
**Religion**					
Islam				1.52[1.34–1.73]	<0.001
Hinduism				1.80[1.52–2.04]	<0.001
Others				Ref	
**Household head**					
Male				1.05[0.95–1.17]	0.352
Female				Ref	
**Number of children**					
No children				0.10[0.09–0.12]	<0.001
1–2 children				0.40[0.36–0.44]	<0.001
3 or more children				Ref	
**Family size**					
≤4				0.72[0.67–0.78]	<0.001
>4				Ref	
**Intention of contraceptive use**					
Intends to use				0.72[0.60–0.86]	<0.001
Does not intend to use				Ref	
**Current residence with partner**					
Living with partner				0.90[0.81–0.99]	0.037
Staying elsewhere				Ref	
**First cohabitation age**					
≤19 years				0.85[0.78–0.92]	<0.001
>19 years				Ref	

## Discussion

This study is a large-scale study specifically designed to estimate the prevalence and associated factors of unintended pregnancy in six South Asian countries. Since most of the countries in this sub-continent were fallen under lower- and middle-income countries [8], they contribute a significant portion in unintended pregnancy and most of which end up in life furtive induced abortions [4]. The unintended pregnancy ranged between 11.9% in India and 28.4% in Bangladesh, whereas the overall prevalence is 19.1% among the six South Asian countries. This indicates a larger number of unintended pregnancy for South Asia than previously reported [4]. But the rate of unintended pregnancy was still low in South Asia compared with developed regions with some significant variation among the South Asian countries [4]. A study from Bangladesh using 2011 BDHS data found the prevalence of unintended pregnancy was approximately 28% among the women [12]. This result is supported by our study using BDHS 2014 dataset. Our study showed increased rate of unintended pregnancy among Indian women compared to a previous study [23].

The multivariate analysis revealed that Bangladeshi women have higher chances of experiencing unintended pregnancies, compared to women in India followed by Nepal, Maldives and Pakistan. The possible reason may be most people lives in village territory, and they are not much aware of family planning. Also,unavailability and less knowledge about contraceptive use, and inadequate health care service are responsible behind unintended pregnancy [12]. Our study also found that women aged 15–19 years were more likely to experience unintended pregnancy compared to women in other age categories. A research conducted in Pakistan found that women aged below 20 years had the highest risk of unintended pregnancy [5]. In general, younger women have higher fertility, higher frequency of sexual intercourse, feel shy to take advice about family planning from relatives or family care organizations, have higher contraceptive failure relative to older women and misunderstanding about contraceptive use, which contributes to unintended pregnancies [5,39,40]. The findings of this study are opposite to a study conducted in sub-Saharan Africa [6] and one in the rural area in Bangladesh [41]. The prevalence and odds of unintended pregnancy of urban women were lower than rural among the six South Asian countries. This evidence coincides with other studies [4,5]. This difference may be due to the difference in sociodemographic characteristics of the people in these regions.

Mostly urban women are well educated and have better knowledge of emergency contraceptive use and family planning. The opportunity of attending family planning-related programs is higher for urban women. The prevalence of unintended pregnancy is lower for urban women, as they are likely to bear a small number of children [42]. The cohabitation rate of urban women is more moderate than rural, as most of the urban men and women are engaged with outdoor work compared than rural women [43]. However, a previous study in Pakistan reported no statistically significant association between unintended pregnancy and wealth index [5]. But several authors also reported that the wealth index of women was related with unintended pregnancy [41,44,45] and this study likewise found that women in the lowest wealth quintile experienced higher odds of unintended pregnancy than women with the highest wealth quintile [6]. An opposite result was found in sub-Saharan Africa [6]. Rural women are more like to have lower wealth index [46] and their knowledge about emergency contraceptive use is also little [47] which may increase the prevalence rate of unintended pregnancy [48]. Notably, this study found most of the women in South Asia were Muslims with higher unintended pregnancy experience like in Sub-Saharan Africa [6]. Overall, Hindu women had the highest risk of unintended pregnancy in South Asia than other religions, and a coincide similar findings were reported in India [49]. In previous studies, unintended pregnancy was more common in Muslim women compared with non-Muslims. One of the main reasons was that Muslim women’s activities were restricted than some other religions [50] and also they were likely to accept pregnancy as “given by Allah”. This conception has no existence in this modern era among Muslim women. So, further research is required about the religious prospect that contributes to this higher prevalence or odds of unintended pregnancy among women globally, considering a limited investigation.

Similar to previous studies [5,8,49], this study confirmed the likelihood of higher unintended pregnancy among women with more children. Small family size obstacles the rate of unintended pregnancy. A few studies confirmed that the likelihood of experiencing unintended pregnancy among women who had more children is higher [51,52]. Women who intended to use contraceptive were less likely to experience an unintended pregnancy. A few studies conducted in sub-Sharan African and central India supported these findings [6,53]. Intended contraceptive use greatly influence to reduce the rate of unintended pregnancies, as any contraceptive can prevent the pregnancy.

We noted that the likelihood of unintended pregnancy was lower among the women who lived with their partner or husband. The relationship between living with partner or husband and unintended pregnancy of women has not been explained in previous studies. Further research is warranted to investigate the causes of the relationship between living with partner or husband and unintended pregnancy studies in sub-Saharan Africa and throughout the world report almost similar finding to this study [6].

### Strengths and limitations

The strength of this study is the use of data from national representative surveys of six South Asian countries and the pooled-analysis. Nevertheless, this study has some limitations; for example, the data does not allow the foundation of causality of unintended pregnancy in south Asia. Other limitations of this study were the very nature of the subject and method it employed, as observed in all DHS programs. Besides, the variables used in the analysis made it difficult, linking their effect with the outcome variable.

## Conclusions

In South Asia, the prevalence of unintended pregnancy was higher among women having a larger number of children (3 or more) and family member (more than 4), aged, non-educated, Hindu, and poorest women. The determinates of unintended pregnancy are age, residence, wealth index, religion, number of children, family size, intension to contraceptive use, current resident with a partner and first cohabitation age. The current dynamics of unintended pregnancy in South Asia can be reversely changed by national-level family planning, and maternal well being polices, where particular interventions to poor women, rural women and early marriage couple. There is a need to encourage women to use contraceptive methods and delay pregnancies. The Government and health authorities of the considered six South Asian countries should ensure promoting family planning programs and making contraceptives widely available for women.
