# Correction: Prevalence of unintended pregnancy and its associated factors: Evidence from six south Asian countries

**DOI:** 10.1371/journal.pone.0259360

**Published:** 2021-10-26

**Authors:** Md. Alamgir Sarder, Sheikh Mohammed Shariful Islam, Md. Maniruzzaman, Ashis Talukder, Benojir Ahammed

The first and third authors’ names are spelled incorrectly. The correct names are Md. Alamgir Sarder and Md. Maniruzzaman.

Sarder MA, Islam SMS, Maniruzzaman M, Talukder A, Ahammed B (2021) Prevalence of unintended pregnancy and its associated factors: Evidence from six south Asian countries. PLoS ONE 16(2): e0245923. https://doi.org/10.1371/journal.pone.0245923
